# Feasibility of a Mobile-Based System for Unsupervised Monitoring in Parkinson’s Disease

**DOI:** 10.3390/s21154972

**Published:** 2021-07-21

**Authors:** Raquel Bouça-Machado, Filipa Pona-Ferreira, Mariana Leitão, Ana Clemente, Diogo Vila-Viçosa, Linda Azevedo Kauppila, Rui M. Costa, Ricardo Matias, Joaquim J. Ferreira

**Affiliations:** 1 Instituto de Medicina Molecular, 1649-028 Lisbon, Portugal; raquelbouca@gmail.com (R.B.-M.); joaquimjferreira@gmail.com (J.J.F.); 2 CNS—Campus Neurológico, 2560-280 Torres Vedras, Portugal; filipaponaferreira@campus.ul.pt (F.P.-F.); marianaleitao.ft@gmail.com (M.L.); linda.m.kauppila@gmail.com (L.A.K.); 3 Kinetikos, 3030-199 Coimbra, Portugal; aclemente@kinetikos.io (A.C.); dvicosa@kinetikos.io (D.V.-V.); 4 Champalimaud Research, Champalimaud Centre for the Unknown, 1400 Lisbon, Portugal; ruimcosta@gmail.com; 5 Zuckerman Mind Brain Behavior Institute, Columbia University, New York, NY 10027, USA; 6 Champalimaud Clinical Centre, Champalimaud Centre for the Unknown, 1400 Lisbon, Portugal; 7 Human Movement Analysis Lab., Escola Superior Saúde—Instituto Politécnico de Setúbal, 2910-761 Setubal, Portugal; 8 Laboratory of Clinical Pharmacology and Therapeutics, Faculdade de Medicina, Universidade de Lisboa, 1649-028 Lisbon, Portugal

**Keywords:** Parkinson’s disease, digital health, remote monitoring, sensors, wearable technology

## Abstract

Mobile health (mHealth) has emerged as a potential solution to providing valuable ecological information about the severity and burden of Parkinson’s disease (PD) symptoms in real-life conditions. **Objective**: The objective of our study was to explore the feasibility and usability of an mHealth system for continuous and objective real-life measures of patients’ health and functional mobility, in unsupervised settings. **Methods**: Patients with a clinical diagnosis of PD, who were able to walk unassisted, and had an Android smartphone were included. Patients were asked to answer a daily survey, to perform three weekly active tests, and to perform a monthly in-person clinical assessment. Feasibility and usability were explored as primary and secondary outcomes. An exploratory analysis was performed to investigate the correlation between data from the mKinetikos app and clinical assessments. **Results**: Seventeen participants (85%) completed the study. Sixteen participants (94.1%) showed a medium-to-high level of compliance with the mKinetikos system. A 6-point drop in the total score of the Post-Study System Usability Questionnaire was observed. **Conclusions**: Our results support the feasibility of the mKinetikos system for continuous and objective real-life measures of a patient’s health and functional mobility. The observed correlations of mKinetikos metrics with clinical data seem to suggest that this mHealth solution is a promising tool to support clinical decisions.

## 1. Introduction

Parkinson’s disease (PD) is a complex neurodegenerative disorder, with a multitude of fluctuating and heterogeneous motor and non-motor manifestations [1]. The currently available therapeutic interventions drastically improve symptoms and quality of life of early stage PD [2]. However, after a few years of dopaminergic therapy, patients suffer from motor and non-motor complications, leading to a deterioration in their quality of life, caregiver burden, and an increase in healthcare resource consumption [2].

Currently, the ability to provide optimized and personalized care is based on a clinical interview, diaries, and scales, performed during short in-person meetings, which take place at best every 3 or 6 months [3,4,5]. As Parkinson’s disease is a complex disease with symptoms that vary across the day and with the medication cycle, a patient’s condition during visits may not accurately reflect the degree and nature of their disability, limiting both the clinician’s ability to capture an accurate image of the patient’s health and consequently manage the disease [3]. Moreover, according to the published evidence, the patient’s performance differs substantially when comparing in-clinic (supervised) with real-life (unsupervised) assessments [6].

To overcome these limitations, several studies have been conducted to explore the role of digital measurement tools, including mobile and wearable technologies, and remote monitoring [4,5,7,8]. These allow for: (1) capturing, with higher frequency, the full complexity, and diversity of PD symptoms; (2) provide a more realistic portrayal of patients’ functionality; and (3) enable closer monitoring of the response to therapy [6,7,9,10].

In this context, mobile health (mHealth) technologies, which can collect and connect clinical and non-clinical information to feed the existing health informatics systems (e.g., electronic medical records), seem a valuable solution to address the growing challenges of cost and provision of quality of healthcare [3]. The key feature of mobile phones (i.e., their pervasiveness, portability, ubiquity, and immediacy), make them a very attractive tool not only for improving, in a cost-effective way, continuous monitoring, clinical decision-making, and communication between stakeholders but also to potentiate patient empowerment to self-manage their disease and to reach a larger number of patients [3].

Although mHealth seems a promising new avenue for PD monitoring in a real-life environment, technical and clinical feasibility in real-life conditions remains to be determined [11,12]. Moreover, this type of tool is frequently associated with adherence problems: it is necessary to find solutions that enable its long-term use [13,14].

In this study, we aim to test the feasibility (including patients’ satisfaction, adherence, and compliance) and usability of an mHealth system (mKinetikos) for continuous and objective real-life measures of patients’ health and functional mobility, in unsupervised settings.

## 2. Materials and Methods

### 2.1. Study Design

A prospective feasibility and usability clinical study was conducted.

### 2.2. Objectives

#### 2.2.1. Primary

To test the feasibility (including patients’ satisfaction, adherence, and compliance) of an mHealth system for continuous and objective real-life measures of patients’ health, in unsupervised settings.

#### 2.2.2. Secondary

To test the usability of an mHealth system for continuous and objective real-life measures of patients’ health, in unsupervised settings.

#### 2.2.3. Exploratory Analysis

To correlate the mHealth system metrics, created based on the data collected during the study, with the recommended clinical tools for evaluating changes in patients’ health and functional mobility, to understand their ability to accurately evaluate these constructs and monitor changes over time.

### 2.3. Participants

Study participants were recruited from the CNS (Campus Neurológico Sénior), a tertiary specialized movement disorders center in Portugal. Patients were eligible if they had a diagnosis of PD according to the International Parkinson and Movement Disorder Society criteria, were able to walk unassisted in the OFF phase, had a smartphone compatible with the mKinetikos app (i.e., Android mobile operating system version 7 or higher), were able to understand and comply, and agreed to participate. Exclusion criteria were the presence of a cardiovascular, pulmonary, or musculoskeletal condition that, according to the clinician’s best judgment, affect a patient’s ability to participate in the study, and the inability to correctly respond to the assessment protocol, according to investigators’ best clinical judgment. The study was undertaken with the understanding and written consent of each participant, received approval from the CNS Ethics Committee (Reference 06-2019) and was in compliance with national legislation and the Declaration of Helsinki. Participants were required to agree to all aspects of the study and were able to leave the study at any time.

### 2.4. Study Supplies

The app mKinetikos (Kinetikos, Coimbra, Portugal) provides a platform that pairs a mobile application for continuous patient monitoring in unsupervised settings and a cloud-based dashboard that dynamically displays all collected information and allows clinicians to remotely interact with patients.

- Mobile-based application (mHealth app): It allows for passive long-term unsupervised functional mobility quantification and position tracking outdoors, remote active testing (e.g., 1-min balance test, finger tapping, and a walk test), and on-demand (onetime or regular) self-reported questionnaires to easily quantify and track a user’s progress and treatment response over time. Additionally, the user can manage their medication and use a direct communication channel with their healthcare team. Users receive a weekly report of their functional mobility and their performance in the active tests.
- Online dashboard: It is a web-based dashboard where the clinical team has access to patients’ information and can interact with them.

### 2.5. Assessment Protocol

Patients were assessed at baseline and for seven months (Table 1 and Appendix A).

At baseline, the mKinetikos application was installed and synced with the Kinetikos online dashboard, and all patients were provided with an explanation of how technical support could be reached during the study. Moreover, after the installation, patients performed the active tests, in ON-state medication, in the presence of the team so they could learn how to use the application and perform each of the active tests. They were also instructed to keep the phone on them for as much as possible during the day and to perform the active tests in ON-state medication.

Daily, data on kinematics-based functional mobility features and displacements (quantity, not geographic location) were collected through kinematic algorithms that run continuously and unobtrusively in the background (mKinetikos passive test).

Participants were asked to answer a daily survey including three 5-point Likert scales about their perception of well-being, daily task limitations, and symptom severity. To minimize recall bias, the survey was sent every day at 7:00 p.m. and stayed available for five hours (with a reminder after three hours).

Additionally, every week, at noon of the same weekday, participants were asked to complete a set of three active mKinetikos tests that included a 1-min quiet stance, a 3 × 10-m walk test, and a 1-min finger-tapping test with the dominant hand (the 1-min duration allows for a significant data collection and do not seem to induce significant fatigue) [15,16]. The active tests were released each week at noon to avoid the morning time, which can be difficult for certain patients, but they remained available until the following one was launched, allowing participants to complete the test whenever they wanted, enhancing compliance. Participants received a reminder every 2 days at 2:15 p.m. if they had not yet taken any of the tests for that week.

Participants received a report at the end of each week that included a global score of their performance in the active tests, as well as a summary of their daily survey scores, allowing for a comparison of outcomes over time.

Due to the lockdown caused by the COVID-19 pandemic, in-person assessments from months 4 to 6 had to be canceled. They were replaced by phone assessments that included the assessments that were suitable to be performed at distance (questionnaires). At month 7, respecting all the COVID-19 safety measures, we were able to perform a complete assessment protocol.

### 2.6. Analysis of mKinetikos Data

All reported mKinetikos metrics correspond to averages over the two weeks before each clinical evaluation [23]. Mobile Patient Global Impression (mPGI) metric corresponds to the average score of daily survey answers. From Tapping active tests, we extracted the total number of touches and the maximum distance between two consecutive touches over the duration of the test. From balance active tests, we calculated the centroidal frequency from the spectral density calculated by using the XY projection of the acceleration signal [24]. Regarding displacements, walking minutes were determined by using the ActivityRecognitionClientAPI (https://developers.google.com/android/reference/com/google/android/gms/location/ActivityRecognitionClient (accessed on 20 July 2021)), and distance traveled was calculated from the smartphone GPS (including walk, car, etc.). Moreover, this automatic walking detection is used as a trigger to collect sensor data (accelerometer, gyroscope, and/or magnetometer upon availability) at 100 Hz. The vertical component of the acceleration was calculated by using the available sensors: directly from the acceleration [25], using SAAM algorithm [26] when magnetometer is available and using a Madgwik filter [27] when the three sensors are available. From these unsupervised walking events, we calculated the stride lengths and stance durations [28]. Heel-strikes and toe-offs correspond to alternating minima in the vertical component of the acceleration [29]. Before this evaluation, a low-pass Butterworth at 2 Hz was applied. Stance duration corresponds to the time between a heel-strike and a subsequent toe-off. Stride length was calculated according to Equation (1) of Zhang et al., 2018 [30], which is an empirical equation that depends on the stride frequency and the vertical acceleration variance. The smartphone location is not expected to have a significant impact on the collected gait metrics [31].

### 2.7. Statistical Analysis

Demographic, clinical, feasibility, and usability data were analyzed by using descriptive statistics. Continuous outcomes were presented as a mean ± standard deviation (SD).

The primary outcome was feasibility and included the following:

- Patients’ satisfaction measured through Item 1 of the Post-Study System Usability Questionnaire (PSSUQ) [32];
- Adherence measured through the number of dropouts at the end of the study;
- Average compliance (throughout the whole study) was measured as the average of the following: (1) percent of the daily surveys (2) percent of expected tests performed (weekly active tests), and (3) percent expected medication registration (based on an individual’s medication schedule). Patients were then divided into three groups: “low” (≤0.33), “medium” (0.33–0.66), and “high” (≥0.66) compliances (these compliances correspond to a global compliance throughout the whole study). Moreover, the temporal evolution of this outcome was measured at months 1, 3, and 7 (after one month, immediately before the COVID-19 confinement, and at the end of the study).

The secondary outcomes were usability, measured with PSSUQ and technical feasibility as measured by the percentage of data that were correctly streamed during the 7 months of the study.

As an exploratory analysis, we evaluated the correlation of a set of mKinetikos variables with the clinical outcomes. For this purpose, a linear regression was used to find which variables better correlate with the results of (1) the Timed Up and Go (TUG) test, (2) MDS-UPDRS finger-tapping score (3.4 item), (3) MDS-UPDRS balance score (3.11 and 3.12 items), (4) an estimation of MDS-UPDRS gait and balance score (calculated as the sum of the following items: 3.10 (gait), 3.11 (freezing), 3.12 (postural instability)), (5) Patient Global Impression-Severity (PGI), and (6) Clinician Global Impression-Severity (CGI). After this, the Spearman correlation coefficient was used to estimate the validity of each mKinetikos variable. These variables were based on the data from the active and passive tests and used the following general expression:

mKinetikos_score_1 = A × mKinetikos_variable_1 + B × mKinetikos_variable_2 + C ~ Clinical_outcome_1

where A, B, and C are the fitting parameters. The fitting procedure was performed by using the Levenberg–Marquardt algorithm implemented in SciPy.

All data processing and analyses were performed by using Python 3.7.6. Graphical representations were generated by using gnuplot 5.2. All variations throughout the study were evaluated by using Friedman test (for repeated data) followed by Dunn’s multiple comparisons post hoc test.

## 3. Results

### 3.1. Cohort General Data

Twenty PD patients were assessed for eligibility and included in the study between November and December 2019. The mean age of participants was 60.8 ± 11.2 years, and the number of men was 14 (70%). The average disease duration was 7.7 ± 5.9 years, 40% (*n* = 8) had motor fluctuations, and dyskinesias and freezing were present in 45% (*n* = 9) of the participants. The mean Hoehn and Yahr stage was 2.0 ± 0.5. Patients’ demographics and clinical characteristics at baseline are summarized in Table 2.

### 3.2. Satisfaction

The mean level of satisfaction at the end of the study was 1.5 ± 1.1 (with 1 representing the highest level of satisfaction).

### 3.3. Adherence

Of the 20 patients included, 17 (85%) participants completed the 7 months of the study. The reasons for dropping out were problems with the balance tests, unwillingness to continue using the app, not attending follow-up, and family problems (Table 3).

### 3.4. Compliance

**Figure 1:**
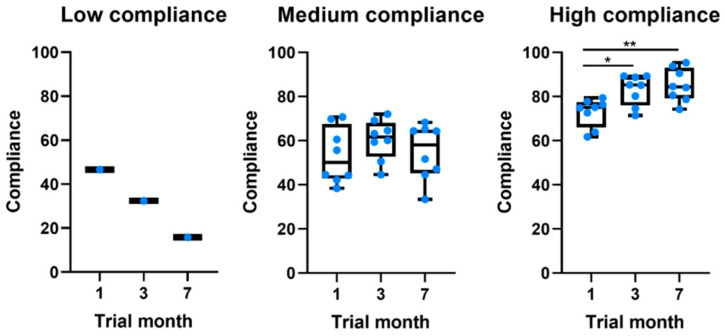
Boxplots comparing the evolution of compliance throughout the study for patients at different levels of global compliance. * *p*-value ≤ 0.05. ** *p*-value ≤ 0.01.

Of the 17 participants in the study, only 5.9% (*n* = 1) showed a low level of global compliance, 47.1% (*n* = 8) a medium level, and 47.1% (*n* = 8) a high level of global compliance to system measures. Table 4 characterizes the sample per level of compliance. When compared to participants with a medium level of compliance, those with a high level of compliance were younger, had a shorter disease duration, and had lower MDS-UPDRS scores. Interestingly, all patients showed moderate compliance values in the first month (Table 3 and Figure 1). Throughout the study, different behaviors were observed. The low-compliance patient gradually stopped using the app. On the other hand, from the other 16 patients, eight kept their compliance constant, whereas the other eight gradually increased their compliance.

**Figure 2:**
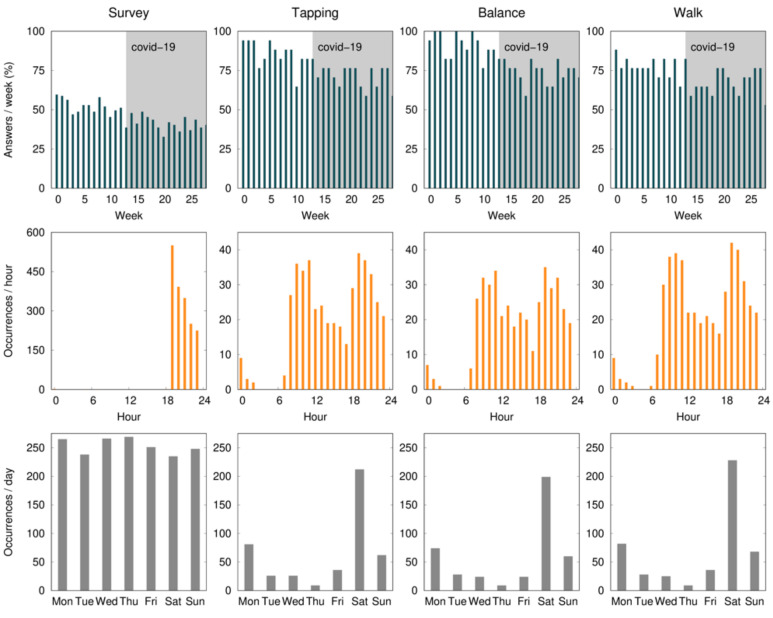
The average percent of compliance (per patient) with the daily survey and active tests (finger tapping, balance, and walk) during the 28 weeks (7 months) of the study, per day of the week, and per hour of the day. Expected data (100%) correspond to one survey per day and one active test per week. The survey notification was sent at 7:00 p.m. and remained available for 5 h. The gray shaded area identifies the COVID-19 confinement phase in Portugal.

The mean percentage of compliance with active tests ranged from 80.9 to 94.1% during the first month, with the finger-tapping test showing the highest and the walk test the lowest level of compliance. During the study, there was a non-significant drop of 19.1% of compliance in the balance and finger-tapping active test, and 11.8% in the walk test. All baseline assessments were performed on a Saturday, and 46.2% of the active tests were performed with a mean delay of 2.5 ± 1.0 days. According to Figure 2, the early hours of the morning and late afternoon are the times that seem the most convenient.

**Figure 3:**
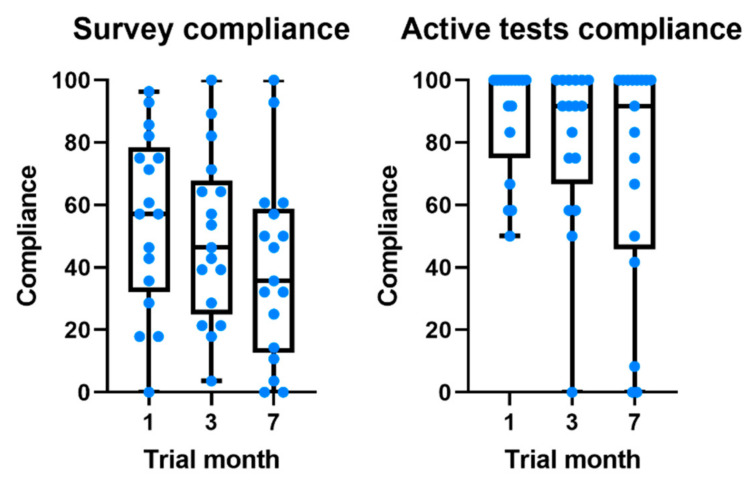
Boxplots comparing the evolution of compliance throughout the study for daily survey and active tests.

Compliance with the daily survey at the end of the first month was 55.5%, registering a non-significant drop of 16% at the end of the study (Figure 3). Compliance with medication alerts was 58.0% at the end of the first month and is also approximately constant during the 7 months of the study (Table 3 and Figure 1). The chat usage was negligible, since most patients opted for direct contact with clinical and technological teams (phone calls and chat applications).

Displacements in months 1 and 3 are similar, which indicates a similar use of the smartphone (for the 7th month, the values are much smaller due to the COVID-19 confinement).

### 3.5. Usability

Generally, PSSUQ showed a high satisfaction with the use of mKinetikos with an average value of 31.3 by the end of the study (scale ranges from 0 to 133, where 0 corresponds to the best system’s usability). Moreover, a 6-point drop in the total score of the PSSUQ was observed throughout the study. In the sub-scales of system usefulness, information quality, and interface quality, the drop ranged from one to two points (Table 3).

We analyzed the items with the worst (highest) score at baseline and at the end of the trial to determine the areas in which participants had the greatest problems at baseline and those that had the most potential for improvement; however, PSSUQ is not validated to be used in this format. The items with a worse mean score (2.6 and 3.7, respectively, in a 7-point Likert scale, where 1 represents “Strongly agree”) at month 1 were related to users’ ability to recover from mistakes by using the system (Item 8) and with the clarity of the information provided (Item 9). At the end of the study, all items maintained or improved the score except for users’ satisfaction with the system interface (Item 10) and their ability to recover from mistakes using the system (Item 8) and to efficiently complete the tasks (Item 7), that worsened 0.2 points.

### 3.6. Technical Feasibility

During the 7 months of the study, 99.9% of the expected data were correctly streamed.

### 3.7. mKinetikos Scores

**Figure 4:**
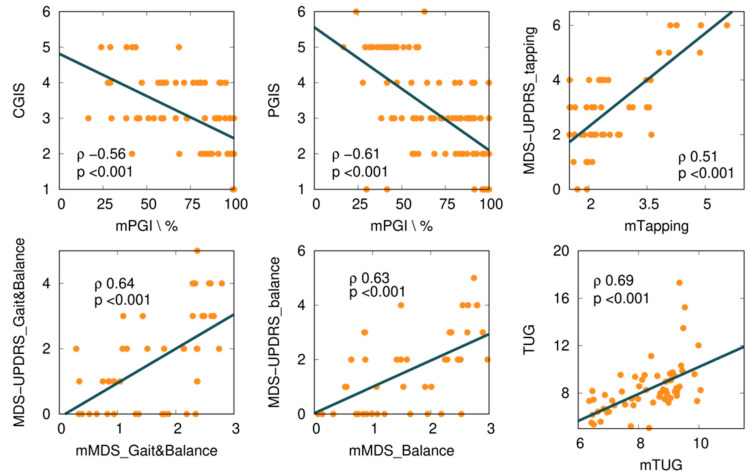
Correlations between mKinetikos scores and the respective clinical outcomes (TUG, MDS-UPDRS finger-tapping, gait and balance, and balance items).

We explored several mKinetikos variables and a combination of variables to predict clinical outcomes. From this approach, we obtained six moderate to strong correlations between mKinetikos metrics and clinical data (Table 5 and Figure 4).

The mTUG showed a strong correlation with the TUG test (r = 0.69, *p* ≤ 0.001). The other mKinetikos scores (mTapping, mMDS_FM, and mMDS_Balance) showed a moderate correlation with the corresponding clinical outcomes, with *r* values ranging from 0.51 to 0.64, *p* ≤ 0.01 (Table 5, Figure 2, and Appendix B).

## 4. Discussion

The present study explores the feasibility and usability of mKinetikos, an mHealth system for continuous unsupervised and objective real-life measures of PD patients’ clinical status. Patients were able to use the mKinetikos app for 7 months, with a high level of satisfaction and compliance. The system also has proved to have good technological feasibility, with 99.9% of the data correctly streamed to the system and a high level of user usability. In the United States and Europe, more than 40% of older individuals do not consult a PD specialist or neurologist. These people are at a higher risk of falling, being admitted to a skilled care facility, or dying, according to published research, demanding the development of new, valid, and feasible mHealth monitoring solutions [33,34]. The mKinetikos responds to this need by collecting and merging unsupervised information, at a distance, from different sources (i.e., patient surveys, active tests, and passive data).

### 4.1. Feasibility

Eighty-five percent (*n* = 17) of the 20 participants enrolled, completed the study, having an approximately constant level of satisfaction throughout the study. Taking into account the 7-months follow-up of the study, our dropout rate (15%, *n* = 3) is lower than other previous similar studies that reported 24–39% dropouts, with 6-months of follow-up [5,7,23,35]. Some factors may have contributed to this difference. First, the number of surveys and active tests requested. In our study, participants were asked to answer one daily survey (three questions) and weekly active tests. In other studies, participants were requested to perform active tests three to four times a day, for a month [23]. Moreover, although it is important to have different sources of information, we believe that an mHealth solution’s primary source of data should be passively collected, not only to improve patient adherence but also to provide more ecological knowledge about users’ average results rather than their best. This poses a challenge in terms of data collection and compliance, as continuous data collection is needed. Second, the number of sensors required by the mKinetikos system. According to a review by the Movement Disorders Society Task Force on Technology, the number of sensors needed to accurately monitor PD symptoms can negatively affect patients’ adherence [7]. The fact that mKinetikos only requires the normal use of a mobile phone by the patient may have contributed to the increased adherence. Third, the availability of a weekly report on the patient’s performance, which we believe worked as a motivational factor contributing to maintaining patient adherence over time [36]. In a similar study where participants used the systems for 11.6 days, the author highlights the importance of having motivational aspects (e.g., community interactions and personalized feedback) to deal with longitudinal decline in the use of such technologies [36].

The two main reasons for dropouts in our study were difficulties managing system error and unwillingness to continue using the system. This goes in line with a previous study that suggested that the reasons for dropouts are likely multifactorial, including study fatigue, loss of the novelty aspect, device-specific, and technical issues [5].

The compliance level was approximately constant throughout the study, and higher than levels previously reported in the literature. According to a review on this topic, 26% of smartphone apps are used only once and 74% of apps are not used more than 10 times [7]. The analysis of the demographic and clinical characteristics per level of compliance revealed that participants with a high level of compliance are younger, with shorter disease duration and a lower level of disability. These results are supported by a previous study that states that patients who were more willing to use mHealth systems are younger and better educated [35]. Younger patients are more familiar with technology and are more likely to recognize the benefits of mHealth devices [35]. However, PD is a disease associated with aging. Even a patient who is comfortable with technology may be unable to interpret the information offered, visualize the interface, and/or have the dexterity to manage the app due to difficulties associated with age and more severe stages of disease (cognitive, visual, and motor dexterity impairments). When developing this type of solution, developers should keep this in mind [7,37].

Interestingly, after 7 months, we still observed high values for active tests. The analysis of compliance per active test showed that users comply more with tests like finger-tapping and 1-min balance, rather than with walk tests. The mKinetikos walk test required participants to walk 10 m, which, in addition to requiring greater effort, may not be easy for some patients to perform at home. This can influence the differences between compliance rates in the active tests.

There was also a difference in the compliance levels between the daily survey (55.5% ± 34.0) and weekly active tests (88.2% ± 28.3). Two factors may have contributed to this: (1) the fatigue associated with a daily survey throughout 7 months; and (2) the daily survey was only available for five hours, while the active tests were available until the release of a new one (i.e., for one week). Although the notification of the active tests was sent on the same day of the week and participants were asked to perform the test on the day they became available, 46.2% of the tests were performed with a mean delay of 2.5 ± 1.0 days (Figure 1). This delay did not have a direct impact on our results, since we use the time at which the test was performed as a reference. In a study evaluating the feasibility of digital motor diaries, the average delay of answering was >4 h [8]. The time of the day when the tests are made available may interfere with compliance. According to our results, the early hours of the morning and late afternoon are the times that seem the most convenient (Figure 1).

Regarding the chat usage, most patients contacted the clinical team during the trial. However, as all patients were already used to being in close contact with clinicians, the mKinetikos chat was used less. We think that, in a broader study, where the patients do not have frequent contact with the clinical team, the chat functionality will be much more important. Having more data and easier communication with the patient does not guarantee a higher level of care. Healthcare professionals should learn how to incorporate these data into their daily routines to be a useful resource rather than a burden. Future research should investigate this topic.

Compliance with medication notifications increased over time. According to a previous study, patients with better drug compliance were more willing to use apps [35]. We hypothesize that the medication notifications, besides increasing drug compliance, increase compliance with the app. Even if patients fail to perform the active tests, they start relying on the medication reminders, continuing to use the system and allowing passive data to continue to be collected. Furthermore, they will enable clinicians to better interpret the results of the daily survey, as they will help them to determine if patients were in an “ON” or “OFF” state of medication at the time they filled out the questionnaires.

### 4.2. Usability

According to the results in the PSSUQ, the system has a good level of usability. Moreover, the observed drop in global usability is within the range of the SD, which indicates that this value was approximately constant throughout the study. However, the system interface (Item 10), the clarity of the information provided (Item 9), the resources to recover from mistakes in using the system (Item 8), and the ability to efficiently complete the tasks (Item 7) can be improved. In a previous study [36] in which 61% of participants had difficulties recovering from errors, the authors suggested a better explanation of the functioning of the areas more prone to cause an error and to provide user-friendly tools or additional information so that they can recover by themselves from the error [36]. Since most PD patients are older adults, who are often unfamiliar with technological solutions, these tips to help solve problems in use should be very clear and easy to understand. To avoid usability issues, future technology solutions should be designed with input from patients, caregivers, and healthcare professionals.

### 4.3. mKinetikos Scores Validity

The mKinetikos variables that best correlate with the results of clinical outcomes resulted in the five composed mKinetikos scores: mTapping, mMDS_Gait&Balance, mMDS_Balance, mTUG, and mPGI. We observed a strong correlation between mTUG and the TUG test (r = 0.69, *p* ≤ 0.001) and a moderate correlation between the other mKinetikos scores and the respective clinical outcomes (*r* values ranging from 0.51 to 0.64, *p* ≤ 0.01). It is noteworthy that the strong correlation of mTUG with TUG test was achieved without any active test, as it only depends on gait parameters that are passively obtained (Table 5). Despite the huge advances in technology and the multitude of devices that are currently available, their widespread use continues to be limited due to the need for valid and meaningful algorithms. For this reason, we included this exploratory analysis in our study.

According to the literature, the design of mHealth systems should be driven by global and functional outcomes and be meaningful to patients, rather than disease-specific [3,7]. The focus of mKinetikos on functional mobility tries to respond to this need. The previous literature showed that patients valued functional mobility for being a meaningful outcome that is easy to describe. Health professionals find it a useful outcome to facilitate patients’ abilities to describe their disability and to help clinicians adopt a more patient-centered approach and to provide individualized care [38]. The TUG test is the gold-standard outcome measure to evaluate functional mobility in PD [39].

### 4.4. Limitations of the Study

This study presents two main limitations: the small sample size and the interruption of monthly in-person assessment between months 4 and 6, due to the COVID-19 pandemic. We believe that the more difficult access to healthcare services may have contributed to increasing compliance with the system and may influence the results in favor of patients’ satisfaction. However, we think that the information generated here is useful for clinicians and for guiding future studies on mHealth systems.

## 5. Conclusions

Overall, mKinetikos seems to be a feasible and usable mHealth system for continuous and remote monitoring of PD patients’ functional mobility and global health status. Throughout the 7-month report, users maintained a high level of satisfaction and compliance. The findings suggest that it may be a useful tool for clinicians to better understand patients’ everyday experiences and provide more patient-centered care.

## Figures and Tables

**Table 1:** **Assessment protocol.** MDS-UPDRS-Movement Disorder Society–Unified Parkinson’s Disease Rating Scale total score and score from each subsection; PDQ-39—Parkinson’s Disease Quality of Life Questionnaire; TUG—the Timed Up and Go test; CGI-S/C—Clinical Global Impression of Severity and Change; PGI-S/C—Patient Global Impression of Severity and Change; PSSUQ—Post-Study usability questionnaire. Due to the COVID-19 pandemic lockdown, MDS-UPDRS-Part III, TUG test, and CGI-C were not performed in months 4–6.

	Baseline	Daily	Weekly	Monthly	End of the Study (Seven Months)
**Clinical interview** Demographic and clinical data					
**MDS-UPDRS** [17]					
**MDS-UPDRS–Part III** [17]					
**Hoehn and Yahr stage** [17,18]					
**PDQ-39**					
**TUG** [19,20,21]					
**CGI-S** [22]					
**CGI-C** [22]					
**PGI-S** [22]					
**PGI-C** [22]					
**mKinetikos active tests** 1-min quiet stance, 10 m walk test, finger-tapping test					
**mKinetikos passive tests** Kinematics-based gait and balance features and displacements					
**PSSUQ**					

**Table 2:** Baseline demographic and clinical characterization of a sample. MDS-UPDRS I evaluates the non-motor experiences of daily living, MDS-UPDRS II the motor experiences of daily living, MDS-UPDRS III motor examination, and MDS-UPDRS IV the motor complications.

Baseline Demographic and Clinical Data (*n* = 20)	
Age (mean, SD)	60.8 ± 11.2
Male sex (% (n))	70% (14)
Time since diagnosis (mean, SD)	7.7 ± 5.9
Presence of motor fluctuation (% (n))	40% (8)
Presence of dyskinesias (% (n))	45% (9)
Presence of freezing (% (n))	45% (9)
MDS-UPDRS I (range 0–52)	9.6 ± 6.0
MDS-UPDRS II (range 0–52)	9.7 ± 7.0
MDS-UPDRS III (range, 0–132)	24.9 ± 16.1
MDS-UPDRS IV (range 0–24)	3.0 ± 4.2
MDS-UPDRS Total (range 0–260)	46.8 ± 26.2
Hoehn and Yahr stage (range 1–5)	2.0 ± 0.5
TUG (s)	8.9 ± 2.8
CGI–S (range 0–7)	3.2 ± 1.0
PGI–S (range 0–7)	3.3 ± 1.0
PDQ-39 (Median [Min, Max]; range 0–7)	25 [2, 73]

**Table 3:** Data from follow-up assessments. * Arbitrary units.

Feasibility Data			
	1 Month	3 Months	7 Months
Satisfaction (PSSUQ—Item 1; range 0–7)	2.2 ± 1.2	1.6 ± 1.0	1.5 ± 1.1
Dropouts (%, n)	0% (0)	15% (3)	0% (0)
Compliance (mean %, SD)			
Patients with a low level of compliance	46.6	32.4	15.8
Patients with a medium level of compliance	53.2 ± 12.8	60.4 ± 9.1	54.8 ± 12.5
Patients with a high level of compliance	72.7 ± 6.5	83.0 ± 6.9	85.2 ± 7.5
Weekly active tests completed (mean %, SD)	88.2 ± 28.3	80.4 ± 39.2	71.6 ± 45.8
Weekly walk tests completed (mean %, SD)	80.9 ± 40.0	75.0 ± 43.7	69.1 ± 46.5
Weekly balance tests completed (mean %, SD)	94.1 ± 15.9	86.8 ± 33.6	75.0 ± 44.5
Weekly tapping tests completed (mean %, SD)	89.7 ± 29.1	79.4 ± 40.3	70.6 ± 46.3
Daily PGI survey completed (mean %, SD)	55.5 ± 34.0	49.6 ± 31.2	39.5 ± 33.0
Participants adherence to medication notifications (mean %, SD)	58.0 ± 34.1	76.1 ± 34.9	62.1 ± 38.7
Interactions through the chat (mean (SD))	4 ± 7	1 ± 2	1 ± 1
**Usability Data**			
	**1 Month**	**3 Months**	**7 Months**
PSSUQ-total score (range 0–133)	37.4 ± 17.3	34.5 ± 16.1	31.3 ± 19.0
PSSUQ-system usefulness (range 0–42)	11.8 ± 5.5	10.9 ± 4.5	10.2 ± 5.4
PSSUQ-information quality (range 0–42)	11.8 ± 7.5	10.4 ± 5.9	10.1 ± 7.6
PSSUQ-interface quality (range 0–21)	5.6 ± 2.9	5.7 ± 3.1	4.4 ± 3.4
**Clinical Data**			
	**1 Month**	**3 Months**	**7 Months**
TUG (s)	8.2 ± 2.2	7.8 ± 1.7	9.3 ± 5.2
MDS-UPDRS III (range, 0–132)	29.5 ± 15.7	27.4 ± 16.9	23.4 ± 17.4
CGI–C (range 0–7)	2.9 ± 1.2	3.1 ± 1.0	3.1 ± 0.9
PGI–C (range 0–7)	2.8 ± 1.4	2.5 ± 0.8	3.5 ± 1.3
MDS-UPDRS Tapping	3.18 ± 1.78	3.13 ± 1.71	2.00 ± 1.85
MDS-UPDRS Balance	2.00 ± 1.80	1.44 ± 1.46	1.73 ± 1.49
MDS-UPDRS FM score	2.06 ± 2.08	1.56 ± 1.55	1.73 ± 1.58
**mKinetikos Scores**			
	**1 Month**	**3 Months**	**7 Months**
mTapping (au *)	2.36 ± 1.34	2.10 ± 1.30	1.75 ± 0.77
mTUG (au *)	8.04 ± 1.45	8.10 ± 1.15	8.22 ± 1.35
mMDS FM (au *)	1.87 ± 0.84	1.50 ± 0.91	1.55 ± 0.91
mMDS Balance (au *)	1.94 ± 0.94	1.56 ± 0.94	1.68 ± 0.94
mPGI (au *)	74.81 ± 22.23	72.56 ± 22.77	70.66 ± 31.28
**Displacements**			
	**1 Month**	**3 Month**	**7 Month**
Walking minutes/day (min)	14.9 ± 10.8	18.2 ± 8.6	9.3 ± 5.9
Distance traveled/day (km)	27.99 ± 36.50	28.00 ± 22.80	12.06 ± 15.88

**Table 4:** Sample characterization per level of compliance (average).

	Low Compliance (*n* = 1)	Medium Compliance (*n* = 8)	High Compliance (*n* = 8)
Age (mean, SD)	55	63.5 ± 11.6	58.8 ± 12.3
Male sex (% (n))	0 (0)	75.0 (6)	87.5 (7)
Time since diagnosis (years; mean, SD)	6.0	10.4 ± 5.2	5.0 ± 5.8
MDS-UPDRS III (mean, SD)	18.0	31.6 ± 20.6	23.5 ± 11.6
MDS-UPDRS Total (mean, SD)	51.0	62.1(30.7)	40.5 ± 17.2
TUG (s; mean, SD)	9.5	10.3 ± 3.7	7.8 ± 1.0
PDQ-39 (mean, SD)	36.0	44.2 (19.7)	19.1 (21.6)

**Table 5:** Correlation between clinical and mKinetikos scores.

Clinical Variable	mKinetikos Score	mVariable1	mVariable2	r	*p*-Value	A	B	C
MDS-UPDRS_Tapping score	mTapping	Number of touches	Maximum distance between touches	0.51	<0.001	−0.03	0.78	4.62
TUG (s)	mTUG	Stride length (m)	Stance duration	0.69	<0.001	−7.01	2.35	13.72
MDS-UPDRS_FM score	mMDS_FM	Stride length (m)	Centroidal frequency (Hz)	0.64	<0.001	−5.72	−0.03	7.75
MDS-UPDRS_Balance score	mMDS_Balance	Stride length (m)	Centroidal frequency (Hz)	0.63	<0.001	−5.67	−0.05	7.96
PGI	mPGI	-	-	−0.61	<0.001	-	-	-
CGI	mPGI	-	-	−0.56	<0.001	-	-	-

## Data Availability

The data presented in this study are available on request from the corresponding author.

## Appendix A. Demographic and Clinical Information Collected during the Study

**(1) Informed consent**
Date of inclusion: ______________
Data sign informed consent: ______________
**(2) Eligibility**
Meet eligibility criteria? Yes □; No □;
**(3) Demographic data**
Birth: _____________
Gender: ___________
**(4) Clinical data**
Year of first symptoms: ___________
Year of diagnosis: _______________
First symptoms: _________________
Motor complications: _________________
Comorbidities:
________________________________________________________________________________________________________________________________________________________________________________________________________________
**(5) Medication**
________________________________________________________________________________________________________________________________________________________________________________________________________________
**(6) Clinical assessments**

- The Timed and Go (TUG) test [19,20,21]

Construct assessed: Functional mobility.
Test description: The TUG test is a clinical test where is asked to the participant to rise from a chair, walk three meters at a comfortable and safe pace, turn, and return to the chair.

- Movement Disorder Society-Unified Parkinson’s Disease Rating Scale (MDS-UPDRS) [17,40]

Construct assessed: Motor and non-motor symptoms of PD patients
Test description: The MDS-UPDRS is a four-part rating scale: Part I (non-motor daily living experiences), Part II (motor daily living experiences), Part III (motor examination), and Part IV (motor examination) (motor complications). Part I contains two parts: IA and IB. IA involves a variety of behaviors that the investigator assesses with all relevant information from patients and carers, and IB is completed by the patient with or without the assistance of the caregiver, but independently of the investigator. The rater completes Part III, which contains instructions for the rater to provide or demonstrate to the patient. Part IV contains instructions for the rater as well as information for the patient to read. The rater completes this section, which combines patient-derived data with the rater’s clinical observations and judgments.

- Parkinson’s Disease Questionnaire (PDQ-39) [41]

Construct assessed: Quality of life
Test description: The PDQ-39 is a self-reported questionnaire, with 39 items to evaluate, PD patients’ disease health related quality of life in eight domains.

- Clinical and Patients Global Impression (CGI and PGI) [22]

Construct assessed: Global impression scales
Test description: These are two one-item scales that clinicians (CGI) or patients can fill out (PGI). It checks all aspects of a patient’s health and determines if their clinical state has improved or deteriorated.

- Post-Study System Usability Questionnaire: The PSSUQ [42]

Construct assessed: Usability
Test description: A self-reported questionnaire designed to assess users’ satisfaction with a product at the completion of a study.
**(7) Daily surveys**
Today I felt good during the day
1. Never
2. Rarely
3. Sometimes
4. Often
5. Almost always
Today during the day, I was able to do all the tasks I wanted to do.
1. Never
2. Rarely
3. Sometimes
4. Often
5. Almost always
How do you rate the severity of symptoms today?
1. Absent
2. Very weak
3. Weak
4. Slightly severe
5. Severe
**(8) Weekly active tests** (performed in On-state medication, at the time of the day more convenient to the participant)

- Ten-minute walk test (three trials)

The patient was instructed to mark a 10 m straight path (i.e., without changes of direction) and walk it three times.

- Finger tapping test (1 min)

The patient was asked to tap on the screen during 1 min.

- One-minute quiet stance

The patient was instructed to put the phone in the pocket before and to stay 1 min in quiet stance. The beginning and the end of the test was marked by a sound the smartphone.

## Appendix B. Equations Obtained in the Fitting Procedure

From tapping active test:

mTapping = −0.03 × number of touches + 0.78 × maximum distance between touches + 4.62 ~ MDS-UPDRS_Tapping

From passive walking:

mTUG = −7.01 × stride length + 2.35 × stance duration + 13.72 ~ TUG

From passive walking and balance active test:

mMDS_FM = −5.72 × stride length −0.03 × centroidal frequency + 7.75 ~ MDS-UPDRS_FM

mMDS_Balance = −5.67 × stride length −0.05 × centroidal frequency + 7.96 ~ MDS-UPDRS_Balance
